# Investigating the efficacy of Ginkgo biloba on the cognitive function of patients undergoing treatment with electric shock: a double-blind clinical trial

**DOI:** 10.25122/jml-2021-0262

**Published:** 2022-12

**Authors:** Masoumeh Nazarinasab, Forouzan Behrouzian, Saeedeh Negahban, Amirali Moghadam Sadegh, Elham Zeynali

**Affiliations:** 1 Department of Psychiatry, Golestan Hospital, Ahvaz Jundishapur University of Medical Sciences, Ahvaz, Iran; 2 Student Research Committee, Ahvaz Jundishapur University of Medical Sciences, Ahvaz, Iran

**Keywords:** Ginkgo biloba, cognition, electric shock

## Abstract

Cognitive impairment is common in patients undergoing electroconvulsive therapy (ECT). Researchers are seeking pharmaceutical compounds with low side effects to decrease these outcomes. This study aimed to investigate the effectiveness of Ginkgo biloba therapy on the cognitive function of patients treated with electroshock. In a double-blinded clinical trial, 80 patients with psychiatric disorders who were candidates for ECT in 2019 were randomly assigned to two groups: the intervention group (receiving Ginkgo biloba drug) and the control group (receiving placebo). We used the Mini-Mental State Examination (MMSE) and Wechsler Memory Scale Recipe III (WMS-III) questionnaires to evaluate the efficacy of the drug on the cognitive function at time 0, after 4 sessions and 72 hours post-final session of ECT. The data were analyzed by SPSS version 22, with a significance level of 0.05. Patients' assessment after the intervention showed that the average MMSE scores in the intervention group (28.92±1.04) were statistically higher than in the control group (27.85±1.56). The average scores of the WMS-III in the intervention group and the control group were 97.45±8.04 and 92.00±4.45 after 4 sessions of ECT, and 100.26±8.33 and 92.40±3.92 after the intervention (p=0.001). According to the findings, Gingko biloba increased MMSE and WMS-III scores in older patients (patients over 40 had better scores in both questionnaires, the drug was more effective, and with no side effects).

## INTRODUCTION

Electroconvulsive therapy is one of the important therapeutic methods for patients with severe mental disorders [1]. This treatment is used in patients with depression, acute mania, bipolar disorder, and acute or chronic schizophrenia [2–4]. Based on clinical evidence, the efficacy of electroconvulsive therapy is well established. Recent advances in electroconvulsive therapy are concentrated on enhancing efficacy and minimizing side effects [5].

Headache, muscle pains, nausea, dizziness, and forgetfulness are common complications of electroconvulsive therapy, and according to the commonness of headache and nausea, these could be resolved by taking painkillers and anti-nausea medications. However, memory impairment is the most important and common complication of electroconvulsive therapy [6].

Cognitive aspects that become influenced immediately after the electroshock include orientation, information processing, anterograde and retrograde amnesia, and diminished visual and verbal perception [7]. Anterograde amnesia occurs for the first time at the beginning of treatment and could remain for a month after electroconvulsive therapy courses. Retrograde amnesia occurs for the first time at the beginning of treatment and could remain for six months after the treatment course with electroconvulsive therapy. 75% of patients mention memory impairment as the worst complication of this treatment [7–9]. Ginko biloba leaf extract is used in cerebrovascular diseases, Alzheimer's dementia, and macroangiopathy with a mechanism of inhibition of mitochondrial dysfunction and effect on cellular apoptosis, which leads to a protective role of nerve, cardiovascular and anti-cancer properties [8]. Its active ingredients include flavonoids, terpenoids, and terpene lactone (ginkgolide and bilobalide) [9]. Several clinical trial studies on Ginkgo biloba have demonstrated advancement in cognitive function in treating and preventing Alzheimer's disease [10]. Being free of radical components and having antioxidants plays an important role in the neuroprotectivity of Ginko biloba extract [11]. The role of Ginko biloba as a protective effect on the liver, optic cells of the eye, DNA repair, and antioxidant and anti-inflammatory action has been proved [12].

Ginko biloba is effective on neurotransmitters involved in cognitive function [13], and it affects the cholinergic pathway in cortex areas [14]. Furthermore, it causes an increase in acetylcholine in hippocampal synapses [15] and increases the density of muscarinic receptors in the hippocampus [16]. Ginko biloba is effective in cognitive stages especially working memory, through adjustment of the cholinergic system [17]. Studies reported that the prescription of Ginko biloba is indirectly effective in memory improvement by reactivating the glutaminergic system followed by cholinergic system activation [18].

Although the role of Ginko biloba in memory improvements has been mostly raised in laboratory animals, limited studies were conducted on depression and cognitive disorders in human cases. Given the importance and occurrence of cognitive disorders in patients treated with ECT, the potential properties of the Ginko biloba plant in treating these problems, and the existence of contradictory studies in this field [19, 20], we aimed to investigate the effect of Ginko biloba on cognitive disorders following electroconvulsive therapy.

## MATERIAL AND METHODS

This double-blind clinical trial study was conducted to determine the effectiveness of Ginkgo biloba capsules on cognitive disorders in hospitalized or outpatients treated with electroconvulsive therapy (ECT) referred to the psychiatric ward of Golestan Hospital. The sample size was determined as 40 people for each group and 80 people for the whole study population based on a similar study [21]. The study was a double-blind clinical trial in which the patient and the nurse distributing the drug and placebo were unaware of allocating patients to the intervention and control groups. Inclusion criteria for the study were: age between 18–60, Schizophrenia, schizoaffective, bipolar or major depressive disorders, which two psychiatrists approved based on DSM-5 criterion, 6–12 sessions of bilateral temporal shock determined, hospitalized patients in the psychiatric ward of Golestan hospital who were candidates for electroconvulsive therapy, minimum literacy or ability to read and write, the ability of verbal communication, no mental retardation or serious cognitive impairment.

Exclusion criteria were: epilepsy, coagulation disorders, usage of anticoagulants and anticonvulsants and NSAIDs (non-steroidal anti-inflammatory drugs), alcohol and drug abuse, cases of dementia and delirium, cases of shock therapy during the previous six months, cases receiving less than 6 sessions of shock therapy and MMSE questionnaire scores below 24 and Wechsler scores below 40, cases of mental disability and pregnancy and lactation.

In this study, the clinical demographic questionnaire was completed (Appendix 1) based on the information obtained from the patient and file contents, including age, sex, illness duration, type of psychiatric disease, and inserting the seizure duration in each session.

**Figure 1:**
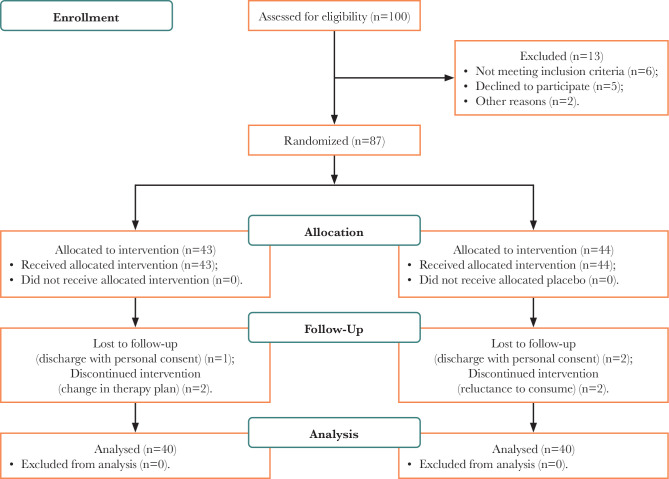
CONSORT 2010 flow diagram.

The patient was assessed using the Mini-Mental State Examination (MMSE) and Wechsler Memory Scale Recipe III (WMS-III). Patients were randomly assigned to two groups of 40 patients (Figure 1). The author provided a 200 mg Ginkgo biloba capsule from Vitarmonil Company of France, financed by the university, and a placebo capsule with a similar shape and color and without a special smell provided by Ahvaz Jundishapur University.

Patients received an average of 2–3 sessions of bilateral ECT per week. The total number of sessions was between 6–12, and during each session, each patient received 50 mg of Succinylcholine, 20 mg of Propofol 1%, 20 mg of lidocaine 1%, and 0.5 mg of Atropine intravenously to induce anesthesia and relax the muscles. Patients were divided randomly into two groups:

1. Intervention group: prescription of Ginko biloba capsule started 48 hours before the beginning of shock therapy. The intervention group was given 6–12 sessions of bilateral ECT (2–3 times per week) and 1 Ginko biloba capsule per day (a 200 mg capsule) after meals until the end of ECT sessions;
2. Control group: received a placebo capsule (looking the same as a Ginko biloba capsule) per day after meal. Patients received the drug until the end of the 6–12 shock therapy sessions.

Patients' cognitive and memory states were assessed using the Mini-Mental State Examination (MMSE) and Wechsler Memory Scale – recipe III (WMS-III) before ECT, after the fourth session, and 72 hours after finishing the ECT course. The questionnaire was completed 24–48 hours after the shock therapy session [22]. The reliability of this test was calculated at 0.78 by Cronbach's alpha method. This questionnaire is scored from 0 to 30. Achieving a score of 27–30 represents a normal cognitive state, 24–26 equals mild cognitive conflict, 18–23 moderate cognitive conflict, and a score less than 18 represents severe cognitive conflict [21]. This scale contains 18 subscales. 11 primary subscales include logical memory – associative forms, verbal pairs – family pictures – letter sequence of numbers – spatial range – auditory recognition, and 7 optional subscales include: information and orientation, vocabulary list, visual reconstruction, mental control, and digit span.

From 11 primary subscales, 8 scores of the index are obtained. Indexes of this scale include: instantaneous auditory, instantaneous visual, instant memory, delayed auditory, delayed visual, delayed auditory recognition, general memory, and active memory, where the total score is obtained from the sum of these indexes. All subscales and indexes had high and acceptable reliability except the faces subscale, which had an alpha of less than 0.70 (0.65).

Mean, standard deviation, and frequency were used for data description [23]. Cohen's d is used when studies report efficacy for continuous measurements, such as a score on a rating scale. To analyze data, an independent t-test and Pearson correlation coefficient were used. The significance level was determined as 0.05. All tests were carried out using SPSS 22 software.

## RESULTS

**Figure 2:**
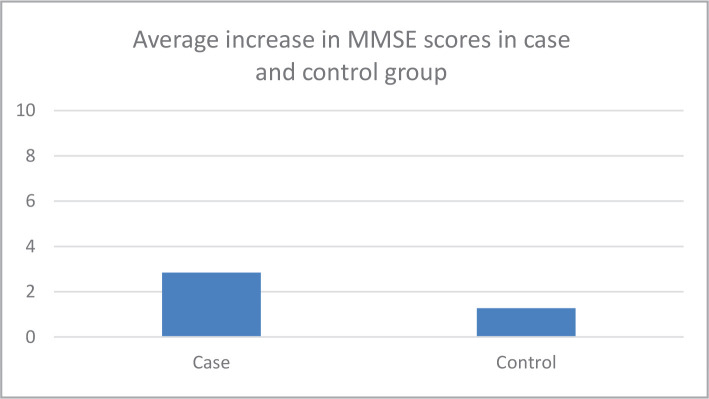
Comparison of average increase in MMSE scores in intervention and control groups.

The average age of all study participants was 34.30±9.04 years; 32.97±8.14 years for the intervention group and 35.62±9.78 for the control group. Gender distribution in the intervention group was 27.5% female and 72.5% male, and 37.5% female and 62.5% male in the control group. 75.0% of participants were single and 25% were married in the intervention group, and 62.5% were single and 37.5% were married in the control group. The type of psychiatric disorders in the intervention group was: 5.83% unspecified psychotic disorder, 37.5% schizophrenia, 10.0% major depressive disorder, 32.5% bipolar disorder. In the control group, 6.6% of participants had unspecified psychotic disorder, 25.0% schizophrenia, 2.5% major depressive disorder, and 55.0% bipolar disorder (Table 1). The average illness duration was 11.57±5.39 years for all study participants, 10.92±5.07 years in the intervention group, and 12.22±5.69 years in the control group. A comparison of the average increase in MMSE scores of people in the intervention and control groups is shown in Figure 2.

**Table 1:** Demographic characteristics of the study population.

Demographic data/clinical disorder	Control group	Intervention group
**Age**	35.62±9.78	32.97±8.14
**Female**	37.5%	27.5%
**Male**	62.5%	72.5%
**Single**	62.5%	75%
**Married**	37.5%	25%
**Primary school**	27.5%	27.5%
**Frist high school**	27.5%	27.5%
**Secondary high school**	45%	35%
**University degree**	0%	10%
**Schizophrenia disorder**	25%	37.5%
**Unspecified psychotic disorder**	6.6%	5.83%
**Bipolar disorder**	55%	32.5%
**Major depressive disorder**	2.5%	10%

Figure 2 showed that there was a statistically significant increase in the mean scores of MMSE in the intervention group (2.85±1.09) compared to the control group (1.27±1.63) (p=0.001).

**Figure 3:**
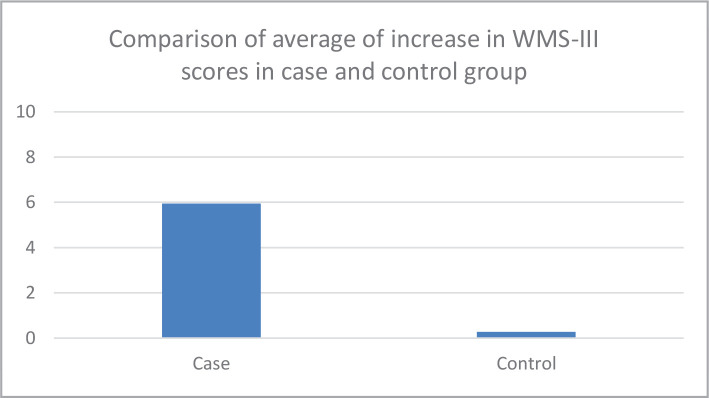
Comparison of the average increase in WMS-III scores in intervention and control group.

The average increase of MMSE scores in patients below the age of forty in the intervention group was 2.70±1.14, and the average increase of MMSE scores in patients aged forty and older in the intervention group was 3.10±0.87. No significant correlation was observed between the age of patients and MMSE scores changes in the intervention group (p=0.26). The average increase of MMSE scores in patients below the age of forty in the control group was 1.31±1.77, and the average increase of MMSE scores in patients aged forty and older in the control group was 1.18±1.25. No significant correlation was observed between the age of patients and MMSE scores changes in the control group (p=0.82). The effect size of MMSE was 1.13. The average increase in WMS-III scores in single patients from the control group was 0.64±1.18, and -0.33±1.23 in married patients. Accordingly, WMS-III scores were significantly higher in single patients than in married patients (p=0.02). A comparison of an average increase in WMS-III scores in the intervention and control groups is represented in Figure 3.

WMS-III scores increased by 3.11±3.67 after the intervention compared to zero time. Separately, the scores increased both in the intervention (6.95±3.02) and the control (0.27±1.28) group. The average increase in WMS-III score in the intervention group was significantly higher than the control group (p=0.001). The effect size of WMS-III was 2.44.

There was a significant difference in the mean scores of WMS-III in patients below the age of forty (5.40±2.87) and patients aged forty and older (7.60±3.01)(p=0.04). However, there was no significant difference in the mean scores of WMS-III in patients below the age of forty (0.17±1.31) and patients aged forty and older (0.54±1.21) in the control group (p=0.4). The mean scores of WMS-III subscales before the intervention are shown in Table 2.

**Table 2:** The means scores of WMS-III subscales at zero time.

Subscale	Group	Number	Mean	Deviation	P-value
**General memory**	Case	40	5.02	.57	0.59
	Control	40	5.10	.67	
**Spatial-temporal orientation (orientation to time and place)**	Case	40	4.57	.54	0.51
	Control	40	4.65	.48	
**Mental control**	Case	40	7.82	.38	0.09
	Control	40	7.65	.53	
**Logical memory**	Case	40	19.92	1.22	0.14
	Control	40	19.47	1.51	
**Digit repetition**	Case	40	17.22	.65	0.27
	Control	40	17.02	.94	
**Visual memory**	Case	40	11.85	1.73	0.7
	Control	40	11.72	1.19	
**Word association**	Case	40	17.72	.90	0.2
	Control	40	17.45	1.01	

Patients in the intervention and control groups had no statistical difference in any subscales before the intervention. The mean scores of WMS-III subscales after 4 sessions of electroshock performed 2–3 times a week are presented in Table 3.

**Table 3:** Mean scores of WMS-III subscales after 4 sessions of electroshock therapy.

Subscale	Group	Number	Mean	Deviation	P-value
**General memory**	Case	40	5.22	.69	0.33
	Control	40	5.07	.69	
**Spatial-temporal orientation (orientation to time and place)**	Case	40	4.67	.47	0.66
	Control	40	4.62	.54	
**Mental control**	Case	40	8.40	.54	0.42
	Control	40	8.27	.81	
**Logical memory**	Case	40	21.27	1.19	0.001
	Control	40	19.60	1.48	
**Digit repetition**	Case	40	17.62	.74	0.16
	Control	40	17.32	1.14	
**Visual memory**	Case	40	13.80	.88	0.001
	Control	40	11.67	.79	
**Word association**	Case	40	19.10	1.23	0.001
	Control	40	17.52	.96	

Patients in the intervention and control group had no significant statistical difference in general memory (p=0.33), spatial-temporal orientation (p=0.66), mental control (p=0.42), and digit repetition (p=0.16) scales after 4 sessions of electroshock. However, there were significant differences in the mean scores of logical memory (p=0.001), visual memory (p=0.001), and words association (p=0.001) subscales in the intervention group. The mean scores of WMS-III subscales after the intervention are presented in Table 4.

**Table 4:** The mean scores of WMS-III subscales 72 hours after the end of the last session of electroshock (after the final session).

Subscale	Group	Number	Mean	Deviation	P-value
**General memory**	Case	40	5.32	.72	0.14
	Control	40	5.10	.63	
**Spatial-temporal orientation (orientation to time and place)**	Case	40	4.72	.45	0.34
	Control	40	4.62	.49	
**Mental control**	Case	40	8.80	.56	0.003
	Control	40	8.20	.98	
**Logical memory**	Case	40	22.32	1.40	0.001
	Control	40	19.67	1.47	
**Digit repetition**	Case	40	19.20	.99	0.001
	Control	40	17.45	1.13	
**Visual memory**	Case	40	14.82	.38	0.001
	Control	40	11.70	.93	
**Word association**	Case	40	20.27	1.19	0.001
	Control	40	17.55	1.03	

There were no significant differences in the mean scores of general memory (p=0.14) and spatial-temporal orientation (p=0.34) scales after the intervention in both groups. However, there were significant differences in the mean scores of mental control (p=0.003), logical memory (p=0.001), digit repetition (p=0.001), visual memory (p=0.001), and words association (p=0.001) between the intervention and control groups.

## DISCUSSION

This study aimed to evaluate the effectiveness of the Ginkgo biloba treatment regimen on the cognitive function of patients treated with electric shock in a double-blind clinical trial. According to the findings, patients older than forty had significantly higher WMS-III scores following the consumption of Ginko biloba. In addition, there were no significant differences in the mean scores of WMS-III in the control group after 4 sessions of electroshock compared with zero time, 72 hours after the end of the last electroshock (final) session compared to 4 sessions of electroshock, and generally 72 hours after the end of the last session of electroshock (final) compared to zero time. Additionally, no significant statistical correlation was found between the WMS-III scores of patients with sex, age, illness duration, number of electroconvulsive therapy sessions, and type of disease. Single patients had higher WMS-III scores than married ones. Hence, this study could report the effectiveness of Ginko biloba drug consumption as statistically significant. No clinical side effects were reported during the treatment.

In a study conducted by Stough et al. in 2011 in Australia, it was reported that the positive effect of Ginko biloba on reducing forgetfulness is applied by interfering with neurotransmitters, including a direct effect on cholinergic neurotransmitters [20]. Furthermore, Hassanzadeh et al. in 2012 showed the effectiveness of Ginkgo biloba on various neurotransmitters, such as increasing cerebral dopaminergic activity, 5HT1A and noradrenergic receptor reduction, reversible inhibition of MAO, A, and B, which has mild anti-anxiety and anti-depression effects in a systematic review on the usage of Ginko biloba plant in autism treatment [24].

These findings were also considered and reinforced by researchers in animal studies, and in a study by Yoshitake et al. in 2010 in Sweden, it was reported that this drug was effective on mood and cognitive problems in mice [25]. The effectiveness of Ginkgo biloba in various cases has been shown in recent studies. In 2020, Yu et al. presented the antioxidant and neuroprotective effects of Parkinson's disease as an alternative treatment [26].

Singh et al., in 2019, by proving the antioxidant effect of Ginkgo biloba, recommended its use in Alzheimer's dementia cognitive impairment [27]. In 2017, Yuan et al. demonstrated the potentially beneficial effects of Ginkgo biloba on cognitive function in people with dementia at doses of 200 mg daily for at least 5 months [28]. In 2017, Zuo et al., using the Ginkgo biloba drug mechanism to prevent mitochondrial dysfunction and the effect on cellular apoptosis, affirmed its efficacy in cerebrovascular disease, Alzheimer's, and macroangiopathies [29]. Birks et al. in 2009 showed improved cognitive outcomes and their effects on daily functioning, quality of life, reduction of dependence on others, and acceptance of treatment by people with dementia after taking Ginkgo biloba supplement [30]. In 2019, Chen et al. showed the role of Ginkgo biloba leaf extract in the prevention and treatment of thrombosis and cardiovascular diseases [31]. In 2020, Fang et al. exhibited the role of biological and chemical properties of Ginkgo biloba on antioxidant, antiviral, anti-tumor, anti-inflammatory, immune-regulating, and hepatic protective effects by assessing active biological components of Ginko biloba [32]. Alimoradian et al. showed the beneficial effects of Ginkgo biloba leaf extract on spatial memory impairment and hippocampal neuronal damage caused by streptozotocin-induced diabetes in rats [33]. De Souza et al. (2020) described the preventive role of Ginkgo biloba in oxidative stress-inducing premature aging in body tissues and the incidence of disease and death, which played a significant role in cardiovascular disease and cancer [34].

Other studies, unlike the previous study, found inconsistent findings on the effectiveness of Ginkgo biloba. Dodge et al. (2008), in the United States, reported that the prescription of 40 mg of the drug three times per day was ineffective in disease progression or protection against memory loss [18]. In 2018, Quidel et al. showed the ineffectiveness of Ginkgo biloba leaf extract in treating tinnitus [35]. In one of these studies, Nikfarjam et al. (2012) in Iran studied Ginkgo biloba with a sample size similar to the present study (81 *vs*. 80 patients in the present study) [5]. Although they studied a single type of disease (Major Depressive Disorder), they reported statistically significant results on the effectiveness of Ginkgo biloba on cognitive status and depression in patients. In their study also, MMSE was used as a measurement tool. However, the strength of the present study compared to their study is the use of WMS-III, which led to significant results, especially since the subscales showed that, except for general memory and spatial-temporal orientation in other subscales, drug efficacy was significant. In 2012 in China, Dong et al. presented the effectiveness of Ginkgo biloba in the treatment of mild cognitive impairment by giving a 50 mg tablet three times per day, which improved patients' cognition and increased MMSE scores [36]. Other studies on the efficacy of Ginko biloba on memory loss associated with ECT were for ECT-induced memory deficits [37] and to improve short-term memory losses associated with ECT [38]. Nevertheless, the WMS-III questionnaire was not used in that study. Therefore this study was one of the few examining the effect of Ginkgo biloba on patients undergoing electroconvulsive therapy and using a different tool for assessing the patients. Another strength was that we assessed the correlation between the demographic characteristics and the drug and showed the relationship between aging and increased drug effectiveness, which is a significant achievement. Although it is difficult to generalize the present results to different communities due to the lack of clinical trials in the study of Ginkgo biloba and the probable findings still contradictory, the present study showed that it could significantly increase patients' MMSE and WMS-III scores with no side effects.

The limitations of this study were patients' dissatisfaction with entering the study and patients' exclusion. Furthermore, patients not following the treatment was one of the problems in the research process. Other limitations of the study were the small sample size, the loss of patients due to changes in the treatment plan during treatment, the high cost of Ginkgo biloba capsules, and the impossibility of long-term follow-up of patients.

## CONCLUSION

The results showed that Ginkgo biloba increased the MMSE and WMS-III scores of patients without any side effects and was more effective in patients of older ages. Given that no side effects were reported during the project implementation, Ginkgo biloba seems to be free of side effects to improve cognitive function. However, the above finding should be repeated in larger sample sizes and multicentrally to generalize to more extended communities.

## Personal thanks

The authors of this article thank all the patients participating in the study, Golestan Teaching Hospital and Ahvaz Jondishapur University of Medical Sciences. It is worth mentioning that this article is the result of a dissertation/thesis.
